# Cholera vaccines for the rich, cholera for the poor: While the global cholera vaccine stockpile runs dry, a booming market for high-income countries exemplifies the chasm between commercial interests and global health needs

**DOI:** 10.1371/journal.pgph.0004359

**Published:** 2025-03-24

**Authors:** Adam R. Houston, Stan Houston

**Affiliations:** 1 Centre for Health Law, Policy & Ethics, University of Ottawa, Ottawa, Ontario, Canada; 2 Faculty of Medicine & Dentistry and School of Public Health, University of Alberta, Edmonton, Alberta, Canada; PLOS: Public Library of Science, UNITED STATES OF AMERICA

## Introduction

In October 2024, the global stockpile of oral cholera vaccine (OCV) ran dry yet again, leaving no unallocated doses for new or worsening outbreaks. This underscores the ongoing shortfall of tens of millions of doses, driven by growing need, with both cases and deaths increasing globally in recent years, and lack of supply, with only one manufacturer currently serving the global stockpile. Meanwhile, however, pharmaceutical companies are reaping profits from a booming parallel market for OCV for a much lower-risk population: travelers from high-income countries (HICs). The current situation, where a tourist from a HIC at essentially zero risk of dying from cholera has more ready access to vaccines than a resident of the endemic country they will visit, exemplifies the chasm between commercial priorities and global health needs.

## Stockpiles running short for the poor

Serious supply problems have become the new normal for the global stockpile, which is managed by the International Coordinating Group (ICG). In October 2022, the ICG – comprising the World Health Organization (WHO), International Federation of the Red Cross and Red Crescent Societies (IFRC), UNICEF, and Médecins Sans Frontières (MSF) – made the major decision to suspend the standard two-dose vaccine regimen in response to shortages [1], shifting to a single-dose regimen evidence indicated was less effective for long-term protection [2].

This means the subsequent shortfalls are already premised on half the preferred number of doses, a vivid example of how the market has failed the low and middle-income countries (LMICs) most at risk. Furthermore, at the time of the 2022 shortage, there were two manufacturers supplying the global stockpile, South Korea’s Eubiologics and India-based Shantha Biotechnics (a subsidiary of Sanofi). However, Shantha/Sanofi made the business decision to discontinue production by the end of 2022. While advance notice was provided, no other company stepped in to fill the gap prior to discontinuation. Nor did Shantha/Sanofi delay their discontinuation, despite calls to do so [3], after the supply crisis became apparent.

## Stock market rising on cholera vaccines for the rich

It’s an overstatement, however, to say the commercial pharmaceutical industry has no interest in cholera vaccine. While the global stockpile is woefully undersupplied, another segment of the market is thriving, with new products and massive increases in sales: travel vaccines for HICs.

There are two main cholera vaccines in this market. Dukoral, a combination of killed cholera bacilli and a subunit of the cholera toxin, is currently produced by Valneva. Dukoral was both the first oral cholera vaccine and the first marketed towards travelers, beginning in Sweden in 1991 [4]. In February 2024, Valneva announced Dukoral sales in 2023 had increased by 72%, driven by higher prices and increased travel demand [5]. While Dukoral is WHO-prequalified, making it eligible for UN agency procurement, and is registered in over 60 countries, its single largest market has historically been Canada [6]. Canada is sufficiently appealing that a second cholera vaccine was approved there in 2023; the press release celebrating this product’s launch for “travelers who plan to visit countries where cholera is present” highlighted that “Canadians are passionate about exploring” [7].

This second vaccine is Vaxchora, a single dose oral live attenuated bacteria formulation whose 2016 approval by the US Food and Drug Administration (FDA) made it the first OCV available in the United States [8]. Its FDA approval was not driven by a pressing domestic cholera risk, but the promise of a Priority Review Voucher (PRV), a potentially lucrative incentive intended to encourage the development of novel tools for diseases predominately afflicting LMICs. Unfortunately, the PRV did not spur true innovation in this instance, instead taking advantage of a loophole treating products as innovative if they had never previously received FDA approval, even if they were well-established globally [9]. Vaxchora itself is a version of the CVD 103-HgR vaccine, previously sold in HICs outside the US as Orochol (Europe) and Mutacol (Canada). In a further irony, Orochol/Mutacol had been discontinued years earlier for commercial reasons including the fact that, thanks to mergers and acquisitions, both Orochol/Mutacol and Dukoral were owned at the time by the same company (Crucell), and the better-established Dukoral was more lucrative [10].

Vaxchora entered the US market at a wholesale price of $225USD per dose [11]. Although available in multiple HICs, Vaxchora lacks WHO prequalification, making it ineligible for UN agency procurement, including the global stockpile. Although there are indications it is less effective in populations with previous cholera exposure, its one-dose schedule and efficacy in small children have generated some excitement about its potential role in combatting cholera outbreaks [10]. Indeed, a previous iteration of CVD 103-HgR was actually used in the very first campaign incorporating OCV into the emergency response to an outbreak [12]. A higher dose version of CVD 103-HgR was previously developed for endemic populations, but has yet to be revived [13].

Unpromisingly, in 2023, Vaxchora was acquired by Bavarian Nordic. The company’s third quarter reporting for 2024 shows a 183% increase in Vaxchora revenue over the year before [14]. Bavarian Nordic’s best-known product is its smallpox/mpox vaccine, variously known as Imvamune, Jynneos and Imvavex. Its approach to that vaccine, which has been stockpiled in some HICs for over a decade but was not made available in any African country outside a clinical trial until August 2024, suggests Bavarian Nordic is likely to prioritize profits over public health for Vaxchora as well.

## Most accessible to those who need it least

Compounding the absurdity of the access gap between HICs and LMICs is the fact the average traveler from a HIC is at vanishingly low risk of serious harm from cholera. A literature review failed to find a single clear reference to a fatal case of cholera in a traveler from a HIC, including from groups assumed to be at higher risk like aid workers, in the 21^st^ Century. Unfortunately, commercial interest seems almost inversely proportional to actual risk to personal or public health, with high-income populations seeking to avoid an already unlikely inconvenience abroad prioritized over those at real risk of serious illness or death.

## Conclusion

In the 19^th^ Century, cholera drove the first binding international agreements to tackle an infectious disease. In the 21^st^, the global stockpile has been drastically underserved, and intermittently left barren, during a period where need has increased considerably. In positive news, the global stockpile’s supply forecast is improving, with Eubiologics making efforts to both simplify and expand production, plus expectations that a second supplier (India’s Bharat Biotech) will be stockpile eligible in 2025, hopefully followed by South Africa’s Biovac [15]. Nonetheless, better safeguards are needed to ensure sufficient supply over the long term is not dependent on commercial whims. Cholera vaccine should be treated akin to a public good, not a lucrative commodity. Maintaining an adequate global supply of vaccines that employ existing technology for a well-known disease like cholera is entirely feasible, but requires the international community to dedicate the necessary political commitment and commensurate resources to doing so, with a focus on regions most impacted by the disease, rather than relying on the caprices of a commercial market most inclined to provide vaccines to those who need them the least.
